# Peer pressure and alcohol consumption in adults living in the UK: a systematic qualitative review

**DOI:** 10.1186/s12889-020-09060-2

**Published:** 2020-07-07

**Authors:** Hannah Morris, John Larsen, Emma Catterall, Antony C. Moss, Stephan U. Dombrowski

**Affiliations:** 1 Freelance researcher, Devon, UK; 2 Drinkaware, Devon, UK; 3 Centre for Addictive Behaviours Research, London South Bank University, Devon, UK; 4 Faculty of Kinesiology, University of New Brunswick, 90 Mackay Drive, Fredericton, New Brunswick E3B 5A3 Canada

**Keywords:** Peer pressure, Adults, Alcohol, Qualitative, Evidence synthesis

## Abstract

**Background:**

Peer pressure to drink alcohol may influence excessive alcohol consumption, which can have adverse impacts on health and wellbeing. While peer pressure to drink alcohol is extensively studied among youth, less examination exists among adults. This systematic review examined qualitative research studies which explored the role and concept of peer pressure within the context of alcohol consumption in adults living in the UK.

**Methods:**

Qualitative studies which explored peer pressure within the context of alcohol consumption or alcohol related behaviours and views in adults (age range approximately 18–52 years) living in the UK were included. Systematic searches conducted in Medline, PsycINFO and Web of Science identified 1462 references, of which 13 studies met inclusion criteria. Thematic analysis was conducted.

**Results:**

Five overarching themes were identified. Four of these themes directly address aspects of peer pressure, including: experiences of peer pressure; consequences of peer pressure; strategies to deal with peer pressure; and conditions perceived to affect peer pressure. The fifth overarching theme explains the wider social context influencing peer pressure. Pressure to drink alcohol affects individuals across the life span and can be experienced as overt and aggressive, or subtle and friendly. Those consuming little or no alcohol are more likely to feel overt forms of peer pressure. Some developed strategies to cope with pressure from drinkers. Peer pressure can result in feelings of social isolation, or giving in by consuming alcohol against ones wishes.

**Conclusion:**

Peer pressure to drink alcohol is a complex and multifaceted phenomenon experienced across adulthood requiring better understanding to support initiatives to decrease the impact of pressure-inducing environments and develop strategies to deal with perceived pressure conditions.

**Trial Registration:**

The protocol for this review is registered with PROSPERO (CRD42019122201). Registered 11 February 2019

## Background

Excessive alcohol consumption has adverse impacts on health and wellbeing [1]. The harmful use of alcohol is a component cause of over 200 disease and injury conditions [2] and causes 5.3% of deaths worldwide [3]. Both the volume of alcohol consumption and pattern of drinking affect the level of alcohol-related harm. In the UK, up to one-quarter of adults (18 years or older) reported exceeding the Chief Medical Officers’ low risk drinking guidelines in 2017 [4–8], and among those who drink 27% reported ‘binge’ drinking (i.e. 8 units for men/6 units for women) on their heaviest drinking day in the previous week [9]. The Global Drug Survey found that drinkers in Britain “get drunk” 51 times per year on average – more often than any other of the 30 nations surveyed [10]. While the proportion of drinkers in the UK has declined over the last decade [9], this reduction has not coincided with a reduction in alcohol harm. In 2017, UK alcohol-specific deaths reached the highest level since 2008, with death rates among men twice that of women [11]. In England, alcohol misuse is cited as the biggest risk factor attributable to early mortality, ill-health and disability for those aged 15–49 years [12]. A better understanding of the complex driving forces behind drinking behaviour in the UK is urgently needed to inform successful intervention strategies aimed at reducing alcohol-related harm.

Normative perceptions are a key predictor of alcohol consumption. Several social-cognitive models, such as the theory of planned behaviour [13], include norm and social influence related constructs as explanation for behaviour [14]. Systematic review evidence consistently suggests that norms play a key role in explaining alcohol consumption [15, 16]. For example, in a systematic review of theory of planned behaviour-based studies predicting alcohol consumption, Cooke et al. (2016) report a sample weighted average correlation of r + =.47 between subjective norms and intentions to consume alcohol, a medium to large effect. Intentions in turn had a large-sized relationship with alcohol consumption (r + = .54). The authors note that the strength of relationship between subjective norms and intentions to consume alcohol is larger than norm-intention relationships typically observed for other health behaviours. The consistency found in relationships between norms and intentions underline the social component driving alcohol consumption.

The social context around alcohol shapes and influences alcohol consumption behaviours, and peer pressure can form a part of this social context. Peer pressure can be defined as ‘*any attempt by one or more peers to compel an individual to follow in the decisions or behaviours favoured by the pressuring individual or group*’ [17]. Perceived peer pressure has been shown to increase engagement in risky drinking practices, such as drinking games [18, 19]. Alcohol consumption frequently occurs in contexts where social influence through others may operate and is embedded within many social rituals. Although a shift towards home drinking has occurred recently in the UK [20, 21], this change in drinking context is still amenable to peer influence, as peers might still be present during alcohol consumption. Other social drinking occasions, such as mixed location heavy drinking and going out with friends, represent a fifth of drinking occasions in the UK identified by Ally et al. [22].

The role of peer pressure in influencing alcohol consumption in adults is poorly understood. Previous systematic reviews on the relationship between peer pressure and alcohol consumption have focussed exclusively on adolescents and college or university students; groups often below the legal age to drink, particularly as the majority of studies have been conducted in a US setting [23, 24]. However, perceptions of peer pressure are likely to continue to exist into adulthood. Peer pressure may be especially relevant when individuals are trying to change their past alcohol consumption behaviour, going against established norms and behavioural patterns which have become socially engrained. Ethnographic studies of adults, for example, have shown the importance of peer pressure specifically regarding the tradition of buying ‘rounds’ in the pub [25]. This highlights an important structural aspect to peer pressure, whereby cultural practices around the way alcohol is purchased and provided to individuals may perpetuate certain patterns of drinking within social groups, potentially even if perceived or actual peer pressure disappeared. A more nuanced understanding of peer pressure might inform the development of successful alcohol reduction strategies.

The aim of this systematic review is to examine qualitative research studies which have explored the role and concept of peer pressure within the context of alcohol consumption in adults living in the UK.

## Methods

We systematically reviewed qualitative studies reporting on peer pressure within the context of alcohol consumption or alcohol related behaviours and attitudes in UK adults. The protocol for this review is registered with PROSPERO (CRD42019122201). Our reporting follows the Preferred Reporting Items for Systematic Reviews and Meta-Analyses (PRISMA) guidance.

### Inclusion criteria

This review included qualitative studies which explored peer pressure within the context of alcohol consumption or alcohol related behaviours and views in adults living in the UK. Table 1 details all inclusion criteria.

**Table 1:** Inclusion criteria details

	Definition
**Population**	UK adults. Operationally defined as where at least 50% of participants were aged 18 or over during exposure to peer pressure, and where at least 50% of participants were situated within the UK during exposure to peer pressure.
**Exposure**	Studies which ‘substantially’ explored the effect of peer pressure. Studies were eligible for inclusion if they stated either a specific research question on the role of peer pressure, or provided a specific conclusion on the role of peer pressure. We adopted the definition of peer pressure by Sim & Koh (2003) ‘*Peer pressure is broadly defined as any attempt by one or more peers to compel an individual to follow in the decisions or behaviours favoured by the pressuring individual or group.*’
**Outcome**	Alcohol consumption, drinking related behaviours (e.g. drink-driving, drinking games, pre-partying, purchasing alcohol), values or perceptions towards drinking.
**Study type**	Any primary study using a qualitative study design. Mixed method primary studies were eligible for inclusion as long as findings from qualitative methodology could be extracted separately.

Studies were excluded when examining adults with alcohol dependence, or adults undergoing treatment for alcohol dependence, or if they were not published in English.

#### Search strategy

The basic search strategy was (alcohol* OR drink) AND (peers OR friend*) AND (United Kingdom OR Great Britain) (*see Additional file**1**for full search strategy*). Searches were conducted in January 2019 and limited to a 25-year time frame (January 1994 to January 2019). Searches were conducted in Medline, PsycINFO and Web of Science core collection.

Five key journals were hand searched in January 2019: Addiction; Alcohol and Alcoholism; Addictive Behaviours; Substance Use and Misuse; and Psychology of Addictive Behaviors. All issues published between January 2018 and January 2019 were checked for possibly relevant papers not yet loaded on electronic databases [26].

Google scholar was searched using the search strategy (alcohol OR drink) AND (peers OR friend). Searches were limited to those published on or after January 1994. For each search the first 100 results were reviewed to identify any potentially relevant studies.

The reference lists of all included papers were examined for additional relevant papers, and forward citation searches were also conducted on all included papers.

#### Screening and data extraction

All titles and abstracts were screened independently by two reviewers (EC and HM) with 98% agreement. Full text screening was carried out independently by two reviewers (EC and HM), with 73% agreement. All disagreements were resolved through discussion or arbitration with a third reviewer (JL).

Data extraction was conducted by one reviewer (HM) and checked by a second reviewer (JL). Data for analysis was considered to be text under the heading ‘findings’ or ‘results’ which pertained to peer pressure and alcohol.

#### Quality appraisal

There is currently no consensus on how to best carry out quality appraisal for qualitative systematic reviews [27]. This review used the Critical Appraisal Skills Programme [28] qualitative checklist. The checklist was operationalised so that for each of the ten quality criteria a study could score two points if the criterion was fully met, one point if it was partially met, and zero points if it was not met at all. This provides a possible maximum score of 20. All studies were appraised by one author (HM) and a sample of four studies was checked by a second (EC). Any disagreements between the two study authors were resolved through discussion.

#### Analysis and synthesis

We carried out a thematic synthesis as described by Thomas and Harden [29]. Two reviewers (HM and JL) familiarised themselves with the data through close reading of all the studies. Line by line coding of all data was then carried out by one reviewer (HM) with a sample of three studies also coded by a second reviewer (JL). Codes were discussed and descriptive themes were tentatively developed and discussed with all review authors. Through this process new themes emerged, and other themes merged together, resulting in overarching themes and sub-themes. An organising framework was constructed to visualise the identified themes and their possible relationships.

## Results

### Search results and study selection

**Fig. 1:**
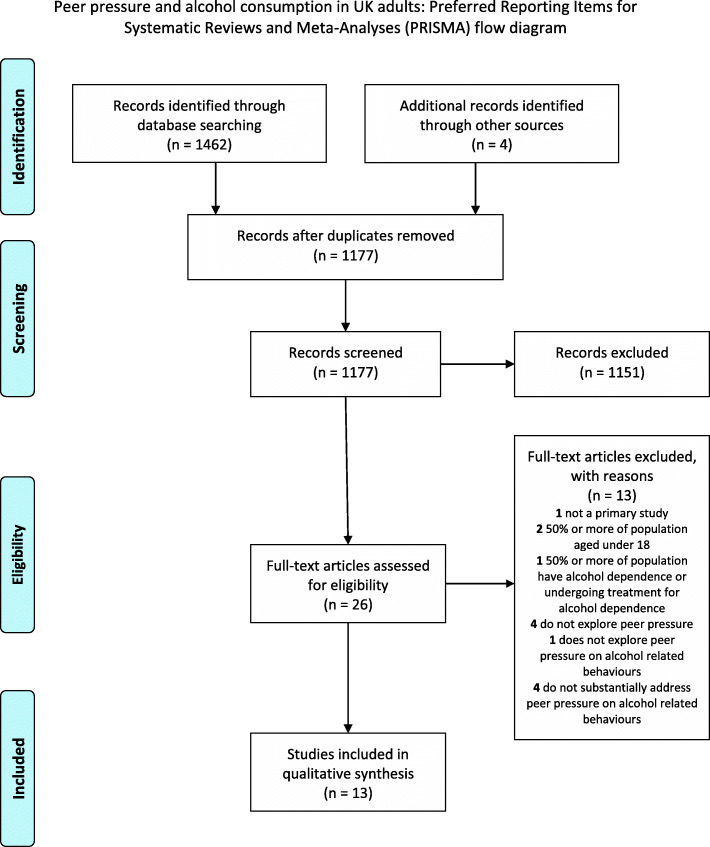
PRISMA flow diagram

The search and study selection process is detailed in the PRISMA flow chart (Fig. 1). The review identified 1462 references through database searching. After screening and full article assessment, 13 studies met inclusion criteria for the review.

### Study characteristics

The 13 included studies were published between 2004 and 2018. Seven of the studies exclusively focused on university students [30–36]. Not all of these studies provided details of the age of participants, but in those studies where age demographics were provided it ranged from 18 to 33 years. Of the remaining studies, all but one focussed on a specific age demographic. Three focused on young people including students [37–39], and two focused on individuals in midlife [40, 41]. Lastly, one study focused on a specific occupation, looking at nightlife entertainers, and these participants were aged 20 to 49 years [42].

Of the seven studies examining university students, the universities were in the following locations: the south of England [32]; East Midlands [34]; West Midlands [35]; North West England [36]; and North England [31]. In addition, one study reported the university was in England [33], and one study recruited from three universities in England and Wales [30]. Of the six non-student studies, three recruited participants from the west of Scotland [40–42], and one each in South East England [37] and South West England [39]. The remaining study reported that the ‘vast majority’ of participants resided in London [38].

The majority of studies (ten out of 13) included both male and female participants. Two studies examined females only [31, 34], and one males only [41]. Four studies examined light or non-drinkers [33, 34, 37, 38], the remaining nine studies included participants with a range of drinking profiles.

Eight studies employed interviews for data collection [32–35, 37–39, 42], three used focus groups [31, 40, 41], one used a narrative question as part of a questionnaire [30], and one used both interviews and a narrative question as part of a questionnaire [36].

The assessed quality of studies ranged from a score of ten to 17 out of a maximum of 20 using the operationalised Critical Appraisal Skills Programme (CASP) checklist. We judged a study as being of higher quality if it scored 15 or more, and lower quality if scoring less than 15. Using this classification, six studies were of higher quality [32–34, 37, 40, 41] and seven were of lower quality [30, 31, 35, 36, 38, 39, 42]. A weight of evidence was then applied using these quality assessments, with greater weight given to studies of higher quality. Table 2 provides further details on study characteristics.

**Table 2:** Characteristics of Primary Studies Included in this Review

Reference; Participants, n; CASP	Participant demographics	Drinking status	Method of data collection
Black & Monrouxe 2014 [30] *n* = 41 *(qualitative data sub-group)* CASP 13	• Students from 3 medical schools in England and Wales • No demographics provided for qualitative sub-group	• No details provided for qualitative sub-group	Questionnaire with qualitative ‘narrative’ question
Carpenter et al. 2008 [31] *n* = 12 CASP 14	• Female students at Leeds University (North England), from a range of academic courses • Aged 18–23 years	• No details provided	2 focus groups
Conroy & de Viser 2012 [32] *n* = 12 CASP 15	• Undergraduate students • Aged 20–29 years • 7 males, 5 females • All from the south of England	• Regular consumers of alcohol	Semi-structured interviews
Conroy & de Viser 2014 [33] *n* = 5 CASP 15	• English university students • Aged 19–22 years	• Non-drinkers (both lifelong non-drinkers and former drinkers (abstinence of ≥6 months))	In-depth, semi-structured interviews
Emslie et al. 2012 [40] *n* = 36 CASP 17	• 15 males, 21 females • Aged 35–50 years (3 younger than 35, 2 over 50) • All respondents were white and lived in the west of Scotland • A socioeconomically diverse sample	• Half (8 males and 11 females) reported drinking over the ‘recommended’ weekly limit (14 and 21 units for women and men respectively). • Six of these could be classed as ‘harmful’ drinkers (over 35 units for women and 50 for men)	8 focus groups
Emslie et al. 2013 [41] *n* = 22 CASP 15	• Males aged between 28 and 52 years (mean 36.9 years) • All were white and lived in the west of Scotland. • Diverse socioeconomic backgrounds	• All drank “regularly”	9 focus groups
Forsyth et al. 2016 [42] *n* = 24 CASP 13	• Entertainers currently working in Glasgow’s pubs and nightclubs (west of Scotland) • 18 males, 6 females • ‘DJs’ (*n* = 8), ‘Band-members’ (*n* = 8) and ‘Variety Acts’ (*n* = 8) • Aged 20–49 years	• No details provided	Qualitative interviews
Graber et al. 2016 [37] *n* = 25 CASP 15	• Young people aged 17–25 years • 13 females, 12 males • Living in South East England • 22 in full time education	• 17 moderate drinkers, 8 non-drinkers	Semi-structured interviews
Herring et al. 2012 *n* = 52 CASP 13	• Young people aged 16–25 years • 26 females, 26 males • 46 students • Vast majority living in London	• 22 current non-drinkers, 30 current light drinkers	Semi-structured interviews
Jacobs et al. 2018 [34] *n* = 8 CASP 16	• Female first year UK undergraduate students at the University of Lincoln (East Midlands) • Aged 18 to 33 years (mean age 21.5 years)	• Non-drinkers (defined as someone who either has never drank alcohol, or has only consumed alcohol once in the previous year)	Semi-structured interviews
MacArthur et al. 2017 [39] *n* = 28 CASP 13	• Young people aged between 18 and 20 years • 13 males, 15 females • Participants lived in both urban and rural environments • Living in South West England • Half of the participants were in employment or seeking employment and half were in, or were planning to attend, tertiary education.	• 13 non-hazardous drinkers - no drinking, or drinking below safe drinking guideline amounts • 14 hazardous drinkers - regularly consuming alcohol over the safe drinking guideline (3–4 units per day for males, 2–3 units per day for females) • 1 harmful drinker – drinking above recommended limits, and at higher levels than most hazardous drinkers	In-depth interviews
Orford et al. 2004 [35] *n* = 11 *(qualitative data sub-group)* CASP 10	• Undergraduate students from a large university in the English West Midlands • Approximately equal numbers of males and females, and of students in each of the three years of study • No age data reported, but all assumed to be adults due to attending university in the UK (where students are 18 + yrs)	• Approximately equal numbers of ‘heavy’ and ‘light’ drinkers	Semi-structured interviews
Piacentini & Banister 2006 [36] Study 1 *n* = 160 Study 2 *n* = 8 CASP 12	**Study 1** • Second year undergraduates at Lancaster University (North West England) undertaking classes in marketing • Aged 19–22 years • 84 females, 74 males, 2 unstated	**Study 1** • Information on drinking status not requested but 4 respondents claimed to be teetotal, over three-quarters of the narratives suggested fairly heavy drinking and the remainder implied light alcohol intake	**Study 1** Written narratives
	**Study 2** • Students at Lancaster University • Aged 19–22 years	**Study 2** • 4 regular drinkers, 4 light/non drinkers	**Study 2** Interviews

Note. *CASP* Critical Appraisal Skills Programme

### Synthesis

**Fig. 2:**
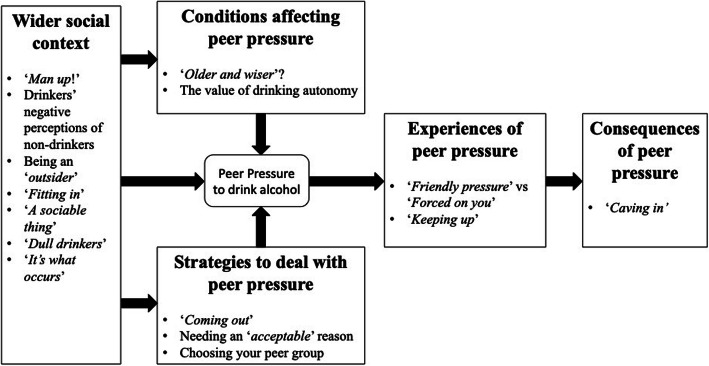
Peer pressure organising framework including overall themes and corresponding sub-themes

Five overarching themes were identified and developed into an organising framework, see Fig. 2. Four of these themes were identified as directly addressing aspects of peer pressure. These were: experiences of peer pressure; consequences of peer pressure; strategies to deal with peer pressure; and conditions perceived to affect peer pressure. The fifth overarching theme provides detail on the wider social context influencing peer pressure. Each of these overarching themes consists of sub-themes derived from the data.

All data from primary study participants is presented in italics and double inverted commas, data from primary study authors is presented in single inverted commas and is not in italics.

### Experiences of peer pressure

Two sub-themes were identified which described elements of the experience of peer pressure.

#### ‘Friendly pressure’ vs. “Forced on you”

Peer pressure to drink alcohol was described differently by participants across studies. A ‘friendly pressure’ to drink was more often expressed by people who themselves were drinkers–this was not typically perceived as peer pressure, but instead a more friendly style of drinking encouragement. However, non-drinkers and some university students experienced a more aggressive form of pressure which was characterised as at times unpleasant and intimidating.

Those describing friendly pressure to drink did not perceive this as peer pressure. As one participant in Graber et al.’s [37] study describes *“When people talk about peer pressure to drink I’m just like ‘doesn’t exist’. I’ve never felt any pressure to, I do it because I’ve chosen to, not because someone’s forced me to [ …][‘peer pressure’] sounds like people are just like ‘Drink drink drink’[ …]I’ve never had someone be like that to me, or[ …]it was only jokingly.”* The study authors note how she describes being ‘jokingly’ egged on, rejecting any notion of peer pressure. A similar type of friendly pressure is also described by a participant in Orford et al.’s study [35], *“If one of us sort of is a bit reluctant then the rest of us will go, ‘Oh go on’, but it doesn’t take much arm-twisting so it’s not really persuasion.”*

In contrast, some participants across studies described experiencing a more ‘forced on you’ and aggressive form of peer pressure. In particular, university students reported to be subject to this kind of pressure. One participant from Black & Monrouxe’s [30] study of university students describes a threatening form of peer pressure to drink. “*Initiation ceremony [ …]2nd years, house. Gang of boys dressed in black bin liners, surrounding freshers and shouting at them to down their drinks. I left, and comforted another fresher who didn’t wish to take part.”* Similar experiences were reported in Jacobs et al. [34] study of non-drinking students, ‘All eight participants interviewed expressed that interactions with drinking students were often unpleasant [ …] Seven out of eight students subject to hurtful comments either to their face, or behind their backs.’ However, this aggressive form of peer pressure was not exclusive to university students, and was also reported in Emslie et al.’s [40] study of individuals in mid-life. ‘(Non-drinkers) described how difficult it was for people to understand and accept that they did not drink alcohol [ …] described receiving a more aggressive reaction [ …] (“*what do you MEAN you don’t drink?*” and “*well, you’ll have one with me!*”)’.

#### “Keeping up”

One form of peer pressure described in several studies was the pressure of ‘keeping up’ with the pace of faster drinkers. The pressure to keep up could be explicit (‘*you get the piss taken out of you if you’re not keeping up with the guys’* [31]) but could also be a part of drinking etiquette – an implicit ‘rule’ of drinking that everyone within the group keeps pace together.

Emslie et al. [41] describes the drinking practice of buying rounds: ‘Buying “*rounds*”—where each man, in turn, bought drinks (usually pints of beer) for the group—was constructed as an essential part of pub etiquette (“*the male equivalent of a friendship bracelet*”), which sometimes led to excessive drinking, due to the pressure to keep up with the fastest drinker.’

A participant in the study by MacArthur et al. [39] describes a less formalised but almost habitual form of “*keeping up”*. *“Sometimes you go out with the wrong frame of mind I suppose and my friend’s bought a pint and then I’ll buy a pint, I’ll sort of drink it and they say they’ve finished theirs and I’m like, oh I’ve got to finish mine. Go and get another pint and I’d try and keep up with them cos they get out more often and I get more drunk than they do.”*

### Consequences of peer pressure

One key sub-theme was identified which explored the consequences of peer pressure.

#### “Caving in”

Some non-drinkers and moderate drinkers reported instances of ‘caving in’ to the peer pressure to drink alcohol. Caving in was reported as a regrettable experience in most cases and was often due to experiencing more aggressive or persistent forms of peer pressure. For non-drinkers, caving in could mean drinking when they did not intend to consume any alcohol, and for moderate drinkers it could mean drinking more than intended.

Jacobs et al. [34] describe how one participant was unable to resist peer pressure, leading her to drink alcohol even though she was against doing so. “*They’d be like, you really do want to though don’t you, they’d pour a drink out for me, sort of say oh just have a drink, [ …]well the second time I went out I sort of caved into that.”* In this instance, the authors note ‘The process of pouring out drinks, an active offer of alcohol, is evidence of direct encouragement, the most direct form of peer pressure.’

Participants in Black & Monrouxe’s study [30] also describe caving in to more persistent and aggressive peer pressure, as illustrated by one student “*I gave up alcohol for lent in my first year at uni. My flatmates forced me to break it. We were pre-drinking in our flat before going clubbing. I was with 6 or 7 friends. I was verbally bullied until I started drinking.”*

However, stronger forms of peer pressure were not always necessary, with some individuals describing difficulties turning down drinks in response to more subtle forms of pressure. An evening entertainer from Forsyth et al.’s study [42] describes *“People keep offering you drinks, particularly if you are playing, and I think if your band-mates are also drinking it’s quite difficult you know to say ‘oh no”’.*

### Strategies to deal with peer pressure

Three sub-themes were identified which address strategies to deal with peer pressure.

#### “Coming out”

Gaining acceptance from peers regarding one’s choices to moderate or abstain from drinking alcohol was rarely reported. Particularly amongst university non-drinkers, the norm appeared to be employing avoidance strategies or excuses rather than “coming out” about ones drinking choices.

Herring et al. [38] observe ‘the extent to which young people were “*open*” about their drinking behaviour, which varied considerably, with some striving to “*blend in*” and not reveal their “*secret*” and others being “*up front*” about their drinking preferences’.

Black & Monrouxe (2014) describe one individual’s strategy to initially join in with the drinking behaviours of their peer group, but once they had been accepted into the group they were able to assert themselves and refute further peer pressure. “*I was encouraged by a group of sports team-mates to down my drink due to losing a drinking game in a sports social with teammates. [ …]It wasn’t particularly pleasant, especially because drinking until drunk is against my religious beliefs so there was a conflict there. However, since then I have managed to draw the line with my team-mates so if I say I’m not drinking any more, then they are OK with that.”*

A similar approach was described by a participant in Conroy & de Visser’s study [33]. “*When first getting to know people it’s important to look like you’ve got a drink. But once you’ve got to know people and they accept it, the best strategy is just to say ‘No thanks’. [ …]So it’s accepted as part of who I am. It’s not a secret, it’s just not something that you broadcast when people who are around you are heavy drinkers.”*

Conroy & de Visser [33] observe a more direct approach was favoured by female participants within their study. “*I say, ‘no, I don’t drink, I never have drunk, I don’t see the reason in drinking, I am not going to drink now.’ They say, ‘just smell it, you’ll like it.’ It’s like, ‘it doesn’t matter if I like it or not, I don’t want to drink.’ I repeat that for a bit and they tend to give up and go away.”* The study authors observe that this participant *‘*preferred to comprehensively refute peer pressure to drink alcohol, choosing to express her behavioural mind-set (“*I don’t”*), its history (“*I haven’t”*) and her stance (“*I don’t see the reason in drinking”*).’

#### Needing an ‘acceptable’ reason

Non-drinkers or moderate drinkers often reported needing an “*acceptable*” reason to give to their peers to explain their non-drinking. This was required to alleviate pressure from peers to drink, to gain acceptance from peers, to avoid losing social status within the group and sometimes also to avoid appearing rude or antisocial.

“*Acceptable*” reasons identified by participants across studies included: detoxing or dieting (females only); being pregnant; driving; unspecified medical reasons; and being on antibiotics. Other strategies described by participants to avoid being detected as a non-drinker included choosing non-alcoholic drinks which looked like alcoholic drinks, for example ‘pretending the bottle of water he needed to avoid dehydration was “*straight vodka”*’ [42], or “*having a half full glass of Coke, that everyone assumes is Coke and Jack Daniels”* [33]. This practice was described as ‘mirroring’ drinks by Herring et al. [38].

Some individuals reported “*nursing*” drinks so that they lasted a long time, buying their own drinks, avoiding being in rounds, and disposing of unwanted drinks.

For other individuals it was easier to avoid situations where the focus was on drinking altogether, as illustrated by a participant in Jacob et al.’s [34] study. “*That’s how people are social. My flatmates would ask me [ …]are you coming out tonight [ …]when I say ‘no, I’ll give this one a miss’, [ …]it makes me feel really antisocial. Every time I say no, it gives off the message that I don’t wanna be social and they’ll stop asking me. If they ask me in the morning [ …]it’ll be ‘I’ll think about it’, then in the evening I’ll be like ‘I’ll have an early night’. I find it quite difficult ‘cause it’s me saying I don’t want to do this with you is being personal.”*

#### Choosing your peer group

For some non-drinkers and moderate drinkers, strategically selecting peers with similar drinking habits, or mixing with peers with a diverse range of consumption levels, was seen as a helpful strategy in avoiding unwanted peer pressure. The importance of supportive peers who understood and respected their decision not to drink were highlighted.

Graber et al. [37] describe how careful selection of a peer group can reduce drinking-related peer pressure. ‘(One participant) related finding an accepting friendship group who ranged from moderate drinkers to abstainers. Knowing other non-drinkers and having peers who know other non-drinkers made her teetotal status less salient.’ This finding is also echoed by Piacentini & Banister [36] ‘Light non-drinkers had a tendency to draw on the ‘seeking social support’ strategy, deliberately seeking the company of other light or nondrinking friends. (One participant) explained how initially she had considered socialising with her university flat mates, but decided against this when she realised the extent of their alcohol consumption. “*I didn’t go out with them. I thought about it at first but when I realised how much they drink [ …]went out with people who don’t drink or drink a little.”’*

### Conditions perceived to affect peer pressure

Two sub-themes discussed conditions which may affect peer pressure.

#### “Older and wiser”

In midlife, Emslie et al. [40] suggest that midlife drinkers’ descriptions initially gave the impression that they experience less peer pressure to drink and are more able to resist peer pressure than their younger selves, by having become “older and wiser”. However, these initial assertions were ‘undermined by drinking stories re-told within friendship groups, jokes which questioned stories of responsible drinking, and accounts of continuing peer pressure to drink’, and it became evident that peer pressure often continues to exist in midlife.

Emslie et al. [40] illustrate the initial presentation by participants that they would no longer be susceptible to peer pressure. ‘He contrasted his wilder younger self with becoming a “*wise old owl*” now: *“[ …]I think we’ve all done that once, hide the drink – get rid of it some way or another, not leave it [because of peer pressure]. But nowadays, you can be honest and say, ‘I’ve had too much – I’ve had enough, and don’t even say to me have another one, because I’m not interested’. I can do that now.”’*

However, as the focus groups progressed, these initial assertions were brought into question. ‘They described how their intention not to drink alcohol – or to stop drinking – was sometimes just not accepted and illustrated this through the repeated chants of the group (e.g. *“go on, go on, go, on, just the one”, “take one, take one”, “just leave the car, just leave the car”* or *“another one for the road”*).’

An “*older and wiser*” theme was also identified in younger drinkers, with evidence that peer pressure diminishes as individuals move through adolescence into young adulthood. However, the drinking behaviour of university students is then described in contrast to this, as evidence that peer pressure may not diminish in young adulthood.

MacArthur et al. [39] observe ‘The influence of peer behaviour diminished somewhat as young people moved through adolescence. Young people still described an influence of their friends, or a more subtle form of influence characterised by “*going along with”* the behaviour of their friends, but young people learnt from their experiences, and felt freer to exert their own choices around drinking behaviour*’.* However, this study also found university students to be particularly vulnerable to peer pressure: ‘Among those who attended university, peer behaviour and local norms again influenced the habitus, but to a greater extent, with young people reporting a clear awareness that drinking was “*the scene”* and integral to university culture. Habitus for these individuals structured more regular and extreme practice reflecting the reported culture of heavy and frequent drinking in these fields and the influence of collective peer behaviour on practice.’

#### The value of drinking autonomy

For some individuals, being a non-drinker or moderate drinker created a strong sense of autonomy and pride at being able to refute peer pressure. “*Making a free choice”* meant feeling like the decisions made about drinking, and while drinking, were truly one’s own. Making a free choice was also experienced as feeling proud about making drinking choices which reflect one’s personality, values and priorities’ [37].

This theme is further illustrated by Herring et al. [38]: ‘Some participants placed great value on being different and not following the “*crowd*” this respondent was proud of being “*different”*: “*I’d say it’s an important part of who I am because it’s always something that I would say I feel slightly, it may be an arrogant thing to say, but I feel slightly proud of not drinking in the face of the fact that I’ve always been pressured to drink by other people.”’*

### Wider social context

Seven sub-themes were identified which describe the wider social context in which peer pressure to drink takes place.

#### “Man up!”

For some male participants, drinking was firmly aligned with masculinity. Challenges to masculine identity provide a basis through which men are able to intimidate each other to drink more. Men wishing to avoid gender-based peer pressure to drink tried to find a way of successfully challenging or circumventing it. Additionally, the need to maintain a masculine, heterosexual identity played an important part in how much an individual drank, how often, and what kinds of drink were consumed.

The quantity, and type, of alcohol consumed, was widely viewed as a strong social marker of gender identity and sexual orientation. This is illustrated in a focus group discussion between two male participants in Emslie et al.’s [40] study. *‘“You walk over with a glass of coke and it’s just [ …]”“Oh! Abuse!” “‘Oh, here comes the gay boy’, do you know what I mean? (laughs)”’.* This observation is further explored in Emslie et al.’s [41] study which included only male participants. ‘Failing to be seen to be drinking like a man was represented as evidence of something being “*wrong*”, which was then associated with being gay or having no money; both appear as reflections of compromised masculinity.’ This was a view held by participants across the reviewed studies, with Conroy & de Visser [32] observing that negative opinions of male non-drinking was ‘a view commonly expressed by participants concerning the risks to men’s perceived masculinity associated with the decision to not drink.’

#### Drinkers’ negative perceptions of non-drinkers

Across a number of studies, non-drinkers reported being subject to negative opinion from drinkers. Non-drinkers discussed how they were stereotyped as “*boring*” by drinkers [30, 34], or perceived as being judgemental [40].

#### Being an “outsider”

Both non-drinkers and drinkers who chose not to drink on certain occasions described feeling like an “*outsider*”. Problems faced by non-drinkers or moderate drinkers included: ‘finding it difficult to get into conversations’ [40]; ‘feeling as though their peers do not want to socialise with them’ [34]; feeling uncomfortable witnessing drunken behaviour [33].

The extent to which drinking alcohol is normalised and expected within UK society was widely commented on across studies. As one young male former drinker explained *“People said these things are normal and everybody is doing it and you’ll be like out of society now”* [38].

#### “Fitting in”

For some participants, drinking and/or getting drunk was an accepted requirement for “fitting in” to a specific social group. In some cases, once individuals had successfully integrated into the group, they could then assert their right not to drink.

Carpenter et al. [31] reported that ‘The first year students in this study stated that they believed that getting drunk would help them to ‘fit in’: “*You are out of your comfort zone. Your friends and family are back home. You will go out more because you have to in order to meet people and then because of that, you end up drinking.”’.*

#### “A sociable thing”

Participants across studies described how drinking was an integral part of socialising, going out and meeting with friends. As one participant in Emslie et al.’s [40] study succinctly put it ‘*“If you don’t go to the pub, you’d never see anyone”’.*

The integral nature of alcohol for socialising was especially pronounced amongst university students. Piacentini & Banister [36] noted, ‘Most participants acknowledged that their social life at university revolved around alcohol consumption. The importance of alcohol in the students’ wider social worlds was clear. “*We all like a drink, it cannot be underestimated for its value in social activities.”‘*.

#### “Dull drinkers”

Moderate and non-drinkers challenged the dominant discourse that drinking is a sociable act. They instead portrayed drinkers as dull, with shallow relationships who limit their social activities to the repetitive act of drinking.

Herring et al. [38] note how the non-drinking participants in their study had to work hard to encourage drinking friends to consider social activities where alcohol was not a central component. ‘In terms of their social lives, young people often encouraged their drinking friends to participate in activities that did not involve alcohol or where alcohol was incidental rather than integral to the event, e.g. to see a film, visit an exhibition. They felt it was too easy for drinkers to ‘default’ to simply going out drinking and not to consider alternatives.’

Emslie et al. [40] describe how non-drinking participants challenged the idea that drinkers were fun and sociable. ‘(Respondents who were non-drinkers) commented on how much interaction in their age group consisted of people talking about going to the pub. They inverted the common cultural portrayal of drinkers as ‘fun’ and non-drinkers as ‘boring’, so that people who did not drink were characterised as entertaining, creative, witty, making real connections with other people and taking responsibility for themselves, while drinkers were portrayed as dull, having repetitive conversations, having shallow relationships with others propped up by alcohol and being irresponsible and unimaginative.’

#### “It’s what occurs”

Across studies, drinking was described as something very normal, which everyone does, and is culturally expected. For many participants it was done without thinking.

Emslie et al. [40] observe that ‘Going out drinking together was widely constructed as the “*natural*” way for men to socialize’. Students in MacArthur et al.’s [39] study also construct alcohol consumption as a normal and almost unthinking act “*I can’t think of a thing that you go out to in the evening except bowling and things like that, where you don’t drink, and even bowling you probably do as well, umm yeah, you just kind of do in the evenings, it’s what occurs.”*.

## Discussion

This study systematically reviewed qualitative evidence on the role and concept of peer pressure within the context of alcohol consumption or alcohol related behaviours in adults living in the UK. This has led to the development of a preliminary framework for understanding peer pressure, across a broader range of people than have been included in previous studies. Findings based on 13 studies highlight the complex nature of peer pressure and the way it operates within the context of alcohol consumption. Peer pressure was perceived across a range of ages and was not solely restricted to adolescents and young adults. Four key aspects of peer pressure were identified: conditions perceived to affect peer pressure; strategies to deal with peer pressure; experiences of peer pressure; and consequences of peer pressure. These four key aspects of peer pressure are further situated within and influenced by the wider societal context.

The findings offer insight into how peer pressure is expressed in adults living in the UK through social norms which influence people’s drinking intentions and drinking behaviours [15]. For example, requirements to ‘keep up’ with a certain level of alcohol consumption expected within a social group and linked e.g. to perceptions of gender identity mean that people in order to ‘fit in’ can drink more than they would have liked to. As suggested by Cooper et al. [43], certain social situations can make it particularly difficult for people to resist social pressure, with student initiation ceremonies among young adults at the extreme end of the scale of social pressure being exercised (e.g., 30), while the pressure is less aggressively expressed, but still present, for example among midlife adults when ‘buying rounds’ in the pub [41]. Being part of a ‘round’ is also an expression of an overt offer, as suggested by Borsari and Carey [23].

This review underlines that peer pressure to drink alcohol forms a social ritual in various UK contexts, which is experienced across a range of ages, and not exclusively in adolescents and young adults. Although some individuals perceived a lessening of peer pressure to drink alcohol over time as they age, accounts suggested a persistent, often subtle perceptions of continuing pressure to drink alcohol.

Individuals across studies report using a variety of strategies to cope with and manage perceptions of peer pressure – whether these involve coming up with an ‘acceptable excuse’, seeking to conceal low/no alcohol consumption or ‘coming out’ to openly state the wish not to drink (at all, or to excess). Interestingly, the review identified how such opposition to peer pressure could be a source of positive identity for the individual, and that friendships could be determined and negotiated based on the non- or low-drinking identity. This is in line with a growing trend of non-drinking among young people [44] and recent societal trends such as (temporal) sobriety [45].

### Suggestions for further research

The current review suggests that further qualitative research is required to understand peer pressure more fully. Despite relatively broad inclusion criteria, only 13 studies could be included. Further qualitative studies focused on peer pressure could build and elaborate on the themes identified in this review (Fig. 1).

Although peer pressure to drink alcohol is experienced across the life span, the majority of reviewed research focussed on adolescents and young adults, typically university students. Further primary research focussing explicitly on adults across a range of ages and socioeconomic backgrounds is urgently required. The experience of peer pressure to drink in non-drinkers and light, moderate and heavy drinkers should be further examined. None of the included studies have focused on peer pressure to drink alcohol across the socioeconomic spectrum. Given the relationship between level of disadvantage and increased alcohol-attributable harm [46], additional research focusing on less privileged populations would be beneficial. It would be particularly interesting, in future work examining these broader demographic groups, to determine whether the framework for different themes which emerged through this review might be a valid framework within which to understand different aspects of peer pressure.

The findings suggest that moderate to heavy drinkers are less likely to perceive peer pressure as something which affects them. Observational and diary-based methods, or other longitudinal methods which allow participants more time to reflect on peer pressure, may be useful methodological approaches to adopt in this population. Unpicking how, and when, adult drinkers acknowledge the influence of peer pressure in their drinking habits maybe a valuable ‘turning point’ which could be harnessed for health promotion strategies. Integrating this qualitative research with the existing quantitative evidence would help target interventions and support for the population segments most vulnerable to this.

The current review has the potential to influence future intervention research in two ways. First, the detailed understanding of the peer pressure process and context provides detail that can inform the selection of appropriate behaviour change techniques [47] and forms of delivery [48] to support change in alcohol consumption as a result of peer pressure. Second, the specific strategies reported by several studies demonstrate that individuals frequently attempt to avoid or manage peer pressure to drink alcohol, and could form the basis for intervention.

### Strengths and limitations

This review systematically examined the qualitative evidence on peer pressure across a range of ages, and provides a comprehensive overview of the best evidence of peer pressure within the context of alcohol consumption or alcohol related behaviours in adults living in the UK. The thematic analysis developed has the potential to inform future research and intervention studies. Some limitations need to be kept in mind when interpreting the findings of this study. The included studies were variable in terms of methodological quality. Although the review focused on adults living in the UK across all ages, the majority of studies included young adults and university students, somewhat limiting the representation from other age groups. Studies that were included did not necessarily focus on peer pressure, but often explore peer pressure as part of a range of factors. Additional and deeper insights into peer pressure might be obtained by research that exclusively focuses on peer pressure.

## Conclusions

Peer pressure to drink alcohol is most commonly associated with adolescents and young adults, but this review found peer pressure affects individuals across the life span. Peer pressure to drink alcohol can take many forms, and may be experienced as overt and aggressive, or subtle and friendly. Non-drinkers are more likely to feel overt forms of peer pressure, whilst heavier drinkers may not identify with peer pressure to drink alcohol, preferring to describe pressure to drink as banter, jokes, and friendly pressure. As a result of the overt pressure to drink, some non-drinkers have developed strategies to cope with pressure from drinkers. Strategies to manage peer pressure worked for some non- or moderate drinkers, for others peer pressure resulted either in feelings of social isolation, or giving into the pressure and consuming alcohol against their wishes.

## Supplementary information

**Additional file 1.** Full database search strategy.

## Data Availability

The datasets used and/or analysed during the current study are available from the corresponding author on reasonable request.

## Abbreviations

CASP: Critical appraisal skills programme
PRISMA: Preferred reporting items for systematic reviews and meta-analyses
PROSPERO: International prospective register of systematic reviews
UK: United Kingdom
