# Supplementary material for: Peer pressure and alcohol consumption in adults living in the UK: a systematic qualitative review
Source: BMC Public Health. 2020 Jul 7;20:1014. doi: 10.1186/s12889-020-09060-2 (PMC7339443; doi:10.1186/s12889-020-09060-2)
Supplement: Supplementary file 1 — Additional file 1. Full database search strategy. [file 12889_2020_9060_MOESM1_ESM.docx]

**Appendix 1**

**Full database search strategy**

|  | | **Medline (via EBSCOhost) 1946 to date** | **psycINFO (via EBSCOhost) 1806 to present** | **Web of Science core collection** |
| --- | --- | --- | --- | --- |
| **Alcohol** | MeSH terms | MM Alcohol Drinking/ | MA alcohols/ | Doesn’t use MeSH terms |
|  |  | MM Alcoholism/ | MA Alcohol drinking patterns/ |  |
|  |  | MM Alcoholic Intoxication/ |  |  |
|  |  | Unqualified search (default: PubMed ID, Registry Number, Registry Word, MeSH Terms, Title, Abstract, Other Abstract, Author, Author Affiliation, Personal Name, Transliterated Title, Contributed indexing fields) | Unqualified search (default: title, Translated Title, Classification Codes, Abstract, Keyword, Subjects, and Author.) | TS=Topic.  (Title, Keyword, and Abstract fields) |
|  |  | alcohol* | alcohol* | alcohol* |
|  |  | drink* | drink* | drink* |
|  |  | drunk* | drunk* | drunk* |
|  |  | booz* | booz* | booz* |
| **Peer pressure** | MeSH terms | MM Exp Peer group/ | MA peer pressure/ | Doesn’t use MeSH terms |
|  |  | MM Exp Social support/ | MA Peers/ |  |
|  |  | MM Exp Interpersonal relations/ | MA Peer relations |  |
|  |  | MM Exp Social environment/ | MA Social support |  |
|  |  | MM Exp Social behaviour/ | MA Significant others |  |
|  |  | MM Exp Peer influence/ | MA Exp Spouses/ |  |
|  |  | Unqualified search (default: PubMed ID, Registry Number, Registry Word, MeSH Terms, Title, Abstract, Other Abstract, Author, Author Affiliation, Personal Name, Transliterated Title, Contributed indexing fields) | Unqualified search (default: title, Translated Title, Classification Codes, Abstract, Keyword, Subjects, and Author.) | TS=Topic.  (Title, Keyword, and Abstract fields) |
|  |  | Peers | Peers | Peers |
|  |  | colleague* | colleague* | colleague* |
|  |  | Friend* | Friend* | Friend* |
|  |  | Spouse | Spouse | Spouse |
|  |  | Family | Family | Family |
| **British** | MeSH terms | Exp United Kingdom/ | None identified | Doesn’t use MeSH terms |
|  |  | TI, AB = title and abstract only | TI, AB = title and abstract only | Ti, Ab = title and abstract only |
|  |  | UK | UK | UK |
|  |  | “United Kingdom” | “United Kingdom” | “United Kingdom” |
|  |  | “great Britain” | “great Britain” | ”great Britain” |
|  |  | GB | GB | GB |
|  |  | Britain | Britain | Britain |
|  |  | England | England | England |
|  |  | Scotland | Scotland | Scotland |
|  |  | Northern Ireland | Northern Ireland | Northern Ireland |
|  |  | Wales | Wales | Wales |

**Limit to:** 1994-2019 (25 years)**;** English language only; Peer reviewed papers only
